# Supplementary material for: Large-scale brain modes reorganize between infant sleep states and carry prognostic information for preterms
Source: Nat Commun. 2019 Jun 13;10:2619. doi: 10.1038/s41467-019-10467-8 (PMC6565810; doi:10.1038/s41467-019-10467-8)
Supplement: Supplementary file 1 — Supplementary Information [file 41467_2019_10467_MOESM1_ESM.pdf]

Supplementary information

Large-scale brain modes reorganize between infant sleep states and carry prognostic information for preterms

Tokariev et al.

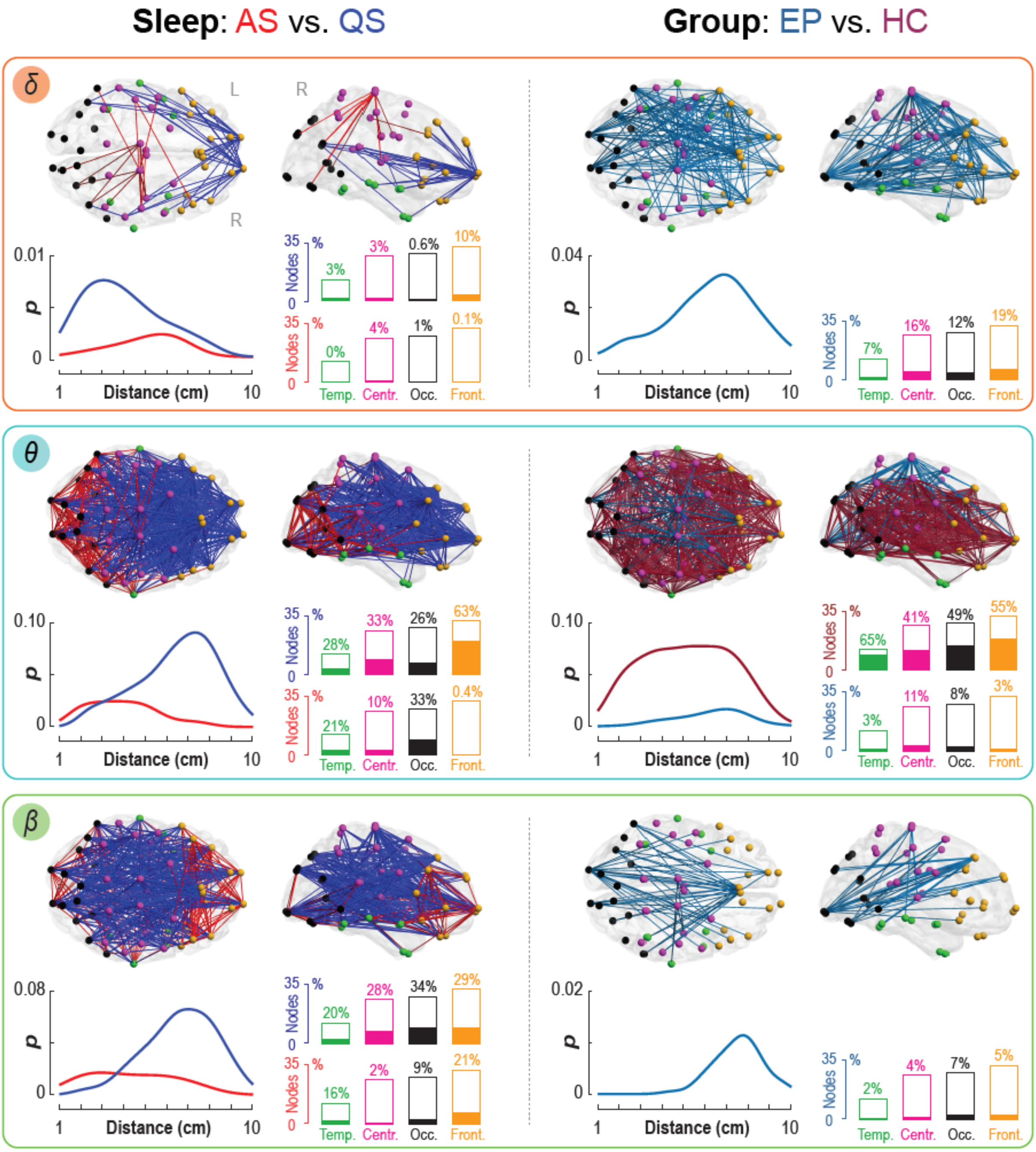

Supplementary Fig. 1. *Changes in cortico-cortical connectivity as a function of sleep state and group (preterm and full-term birth)*. **(Left)** The *main effect of sleep* shows significant broadband connectivity differences in two cortical patterns. The first pattern involves mainly occipital regions (red) whereas the second pattern comprise a widespread set of regions across the brain (blue). *AS* > *QS* (red): delta frequency, p_{FWER} = 0.022; theta and beta, p_{FWER} < 0.0001. *QS* > *AS* (blue): delta frequency, p_{FWER} = 0.003; theta and beta, p_{FWER} < 0.0001. **(Right)** The *main effect of group* shows higher long-range connectivity between remote cortical regions including temporal, central, occipital, and frontal cortices in extremely preterm neonates (EP) compared to healthy controls (HC): delta, theta, and beta frequencies p_{FWER} < 0.0001. In the theta band, HC showed higher connectivity (p_{FWER} = 0.01) among a widespread set of brain regions comprising long-range connections between frontal, central, and occipital regions.

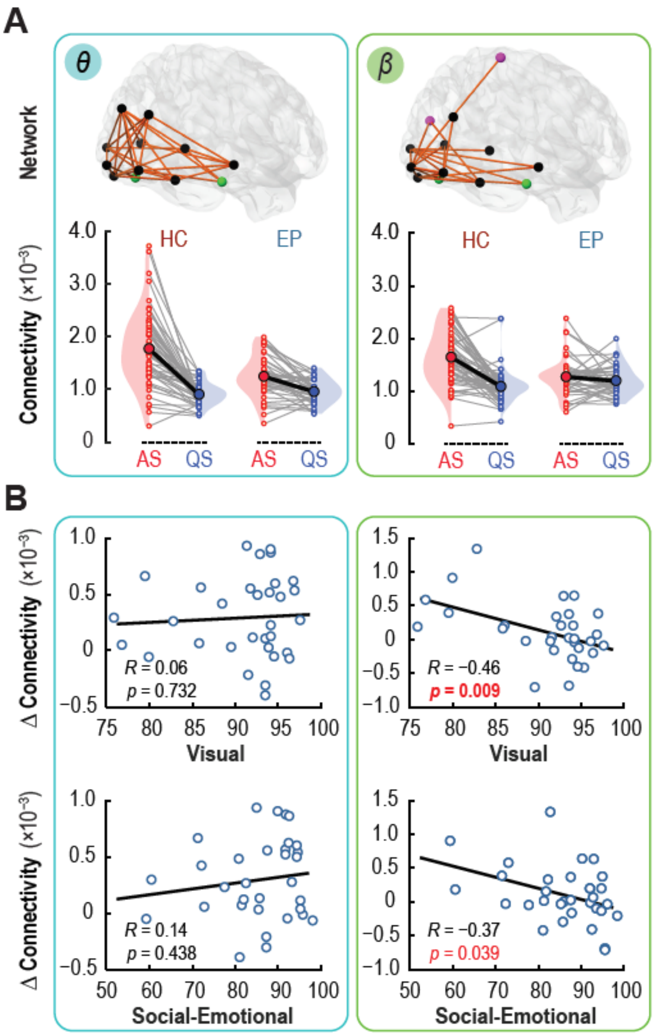

Supplementary Fig. 2. *Group-by-sleep interactions.* **(A)** Significant broadband connectivity differences in overlapping functional networks comprising occipital, temporal and central nodes (theta: p_{FWER} = 0.0018 and beta: p_{FWER} = 0.0046). Such interactions are driven by an attenuated difference in functional connectivity between active (AS) and quiet (QS) sleep in extremely preterm (EP) neonates compared to healthy controls (HC). There were no significant group-by-sleep interactions in the delta band. **(B)** Correlations between changes in the mean cortical connectivity strength of the occipital pattern (connectivity strength of the orange network of panel A in AS minus QS) and neurodevelopmental outcomes assessed at two years of age in EP infants. In EP, partial correlations (two-tailed Pearson) of alpha (**Fig. 4B**) and beta changes in functional connectivity as a function of sleep states highlight strong and significant (FDR corrected; highlighted in red bold) negative associations with visual performance at two years. Similar trends are observed for social-emotional scores at two years in the alpha (**Fig. 4B**) and beta frequency bands but are not significant after FDR correction. Source data are provided as a Source Data file.

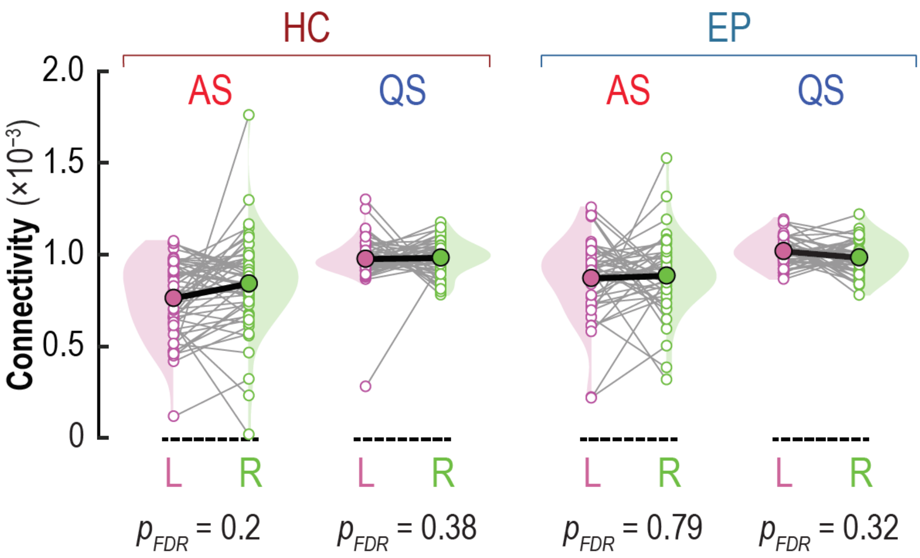

Supplementary Fig. 3. *Lack of left-right asymmetry in functional connectivity patterns.* Formal testing (paired two-tailed Wilcoxon signed-rank test followed by Benjamini-Hochberg FDR correction) of aggregate patterns of functional connectivity within each hemisphere (L = left, R = right) across sleep states (AS = active sleep and QS = quiet sleep) and groups (HC = healthy controls and EP = extremely preterm) did not revealed significant connectivity differences between hemispheres (all $p_{FDR} > 0.2$). Source data are provided as a Source Data file.

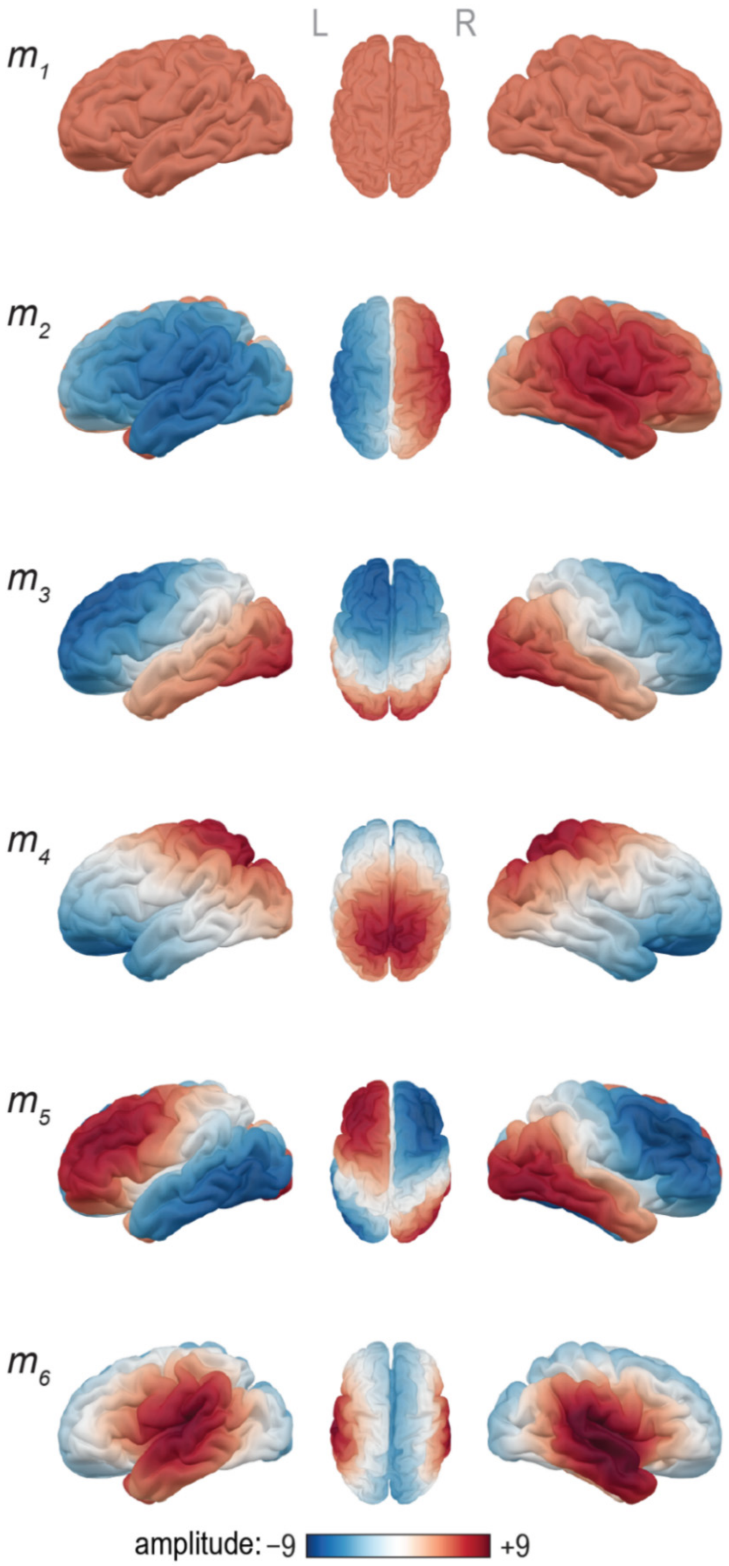

Supplementary Fig. 4. *Eigenmode decomposition.* The first six strongest eigenmodes (m) computed using the geometry of infant cortex at term-equivalent age (Methods). The first global mode (m_1) is uniformly distributed over the cortex and provides the baseline amplitude for global neuronal activity. The second mode (m_2) captures left-right asymmetry in neuronal activity. Modes three (m_3) and four (m_4) together capture anteroposterior and/or dorsoventral activity patterns. More complex patterns of large-scale activity are encapsulated in modes five (m_5) and six (m_6). Colors denote eigenmode amplitudes at each point on the cortex.

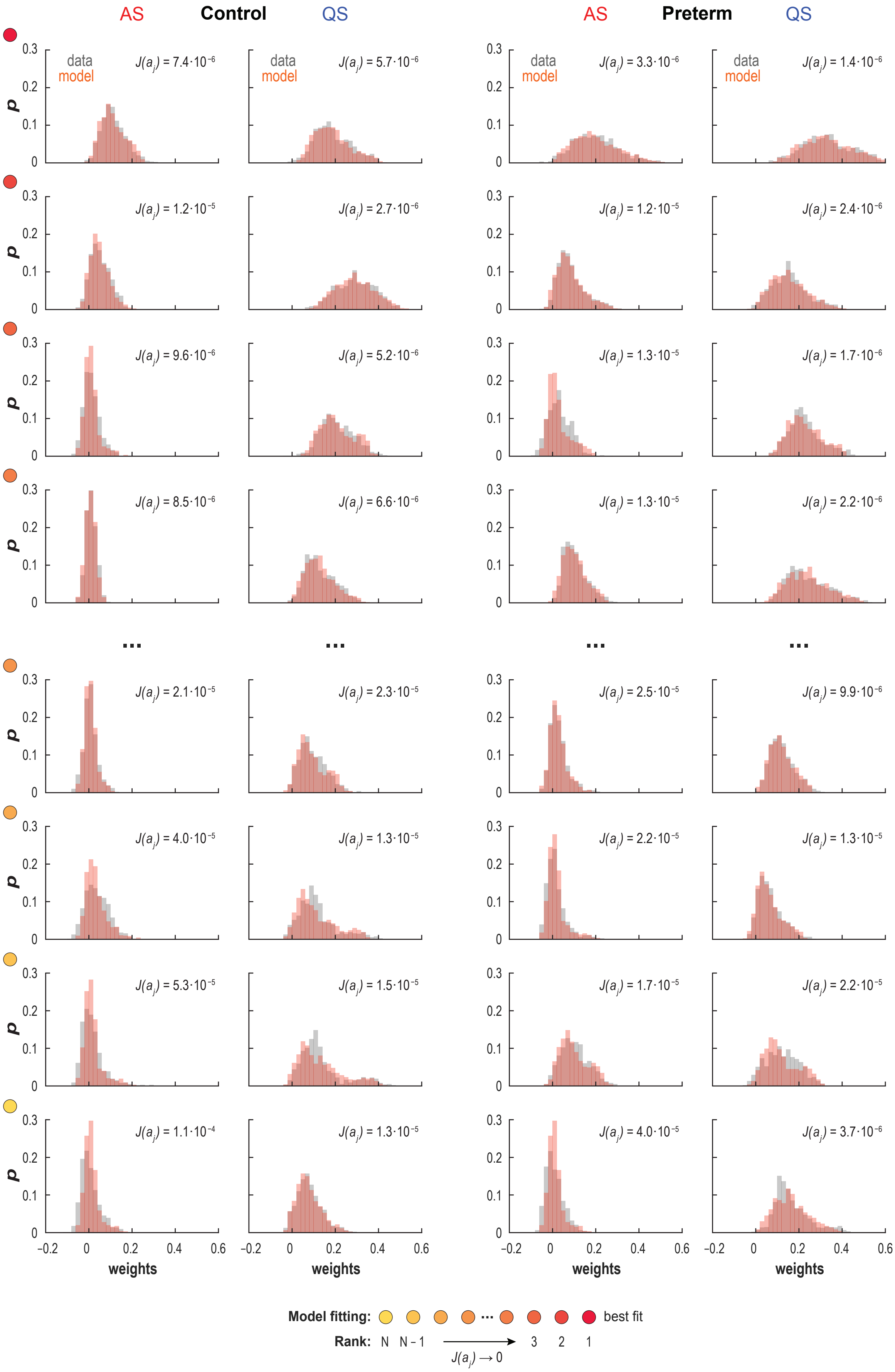

Supplementary Fig. 5. *Exemplar single-subject model fits*. Subjects in each group (controls, left; preterms, right) are ranked according to the best model fit to empirical data averaged across both conditions (AS and QS). Histograms show distribution of *in silico* (orange) and real (gray) functional connectivity values in the alpha frequency range. Better fit corresponds to lower values of the cost function $J(a_j)$ as indic ated by the colored circles.

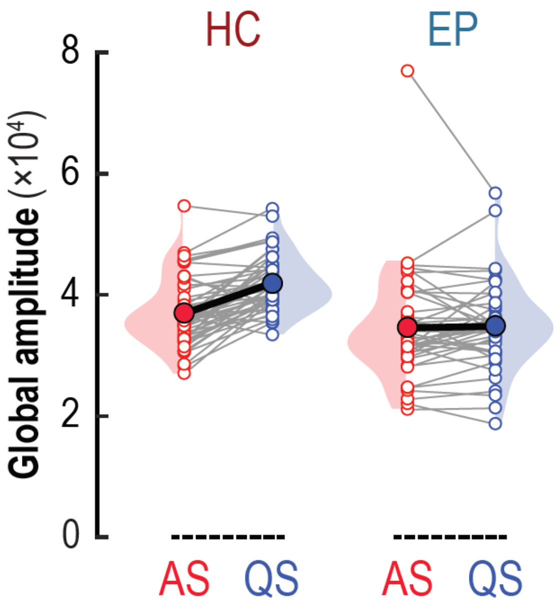

Supplementary Fig. 6. *Empirical changes in global alpha amplitudes as a function of sleep states and group.* Global cortical amplitude was calculated as the mean of the whole-brain amplitude envelopes in the alpha frequency band. The figure shows a significant group (HC = healthy controls and EP = extremely preterm) by sleep (AS = active sleep and QS = quiet sleep) interaction ($F_{1,92} = 21.46$, $p = 1.2{\cdot}10^{-5}$, effect size $\eta^2 = 0.154$, mixed ANOVA). This result is consistent with the changes in energy observed in the first global uniform mode (**Fig. 5C**). Source data are provided as a Source Data file.

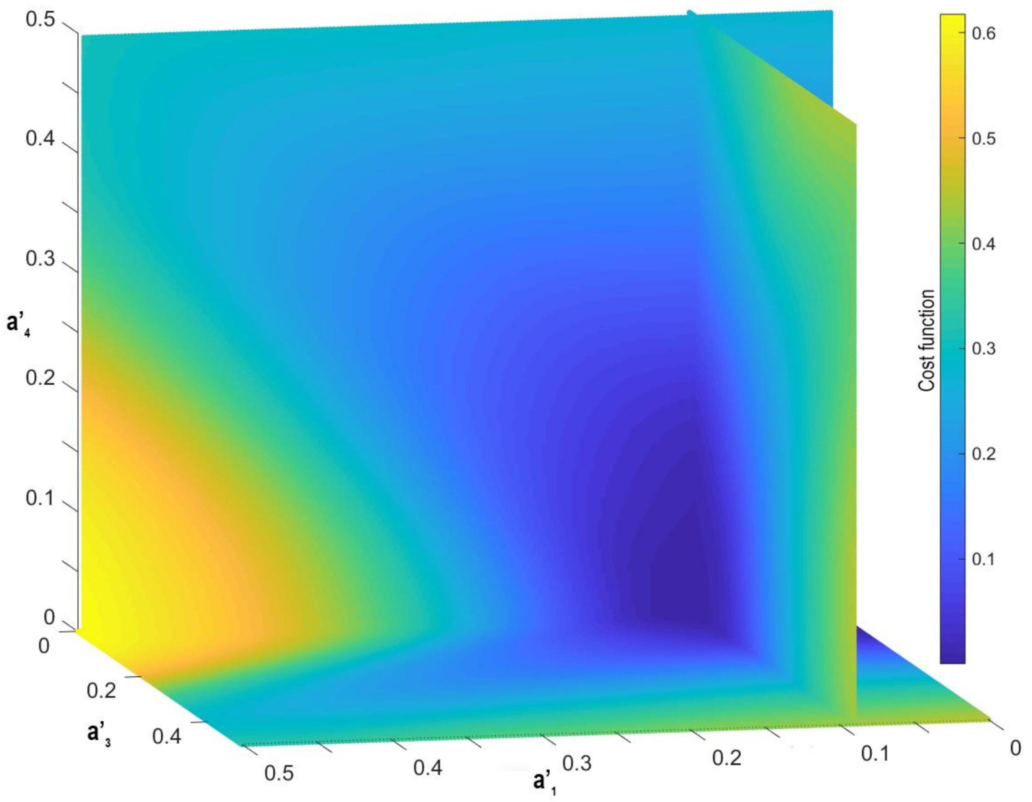

Supplementary Fig. 7. Values of the cost function in the parameter space of mode weights (a'_j, Equation 1) for a representative HC infant in AS (also used for **Fig. 5B**). The mode weights (a'_1, a'_3, a'_4) were calculated between 0 to 0.5, with steps of 0.0025. The figure reveals the smooth and relatively steep change in cost function values, with a unique global minimum.

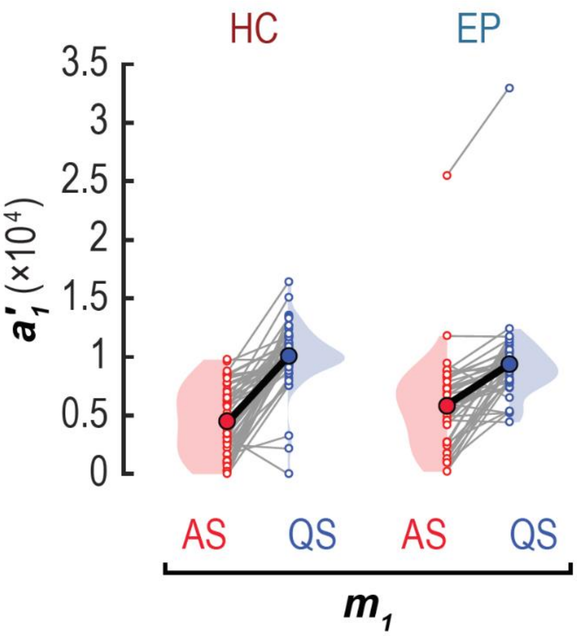

Supplementary Fig. 8. Weights (a_1') of global mode (m_1) including outlier subject omitted on **Fig. 5C** for visualization purposes (note, that all infants were used in the statistical analysis).

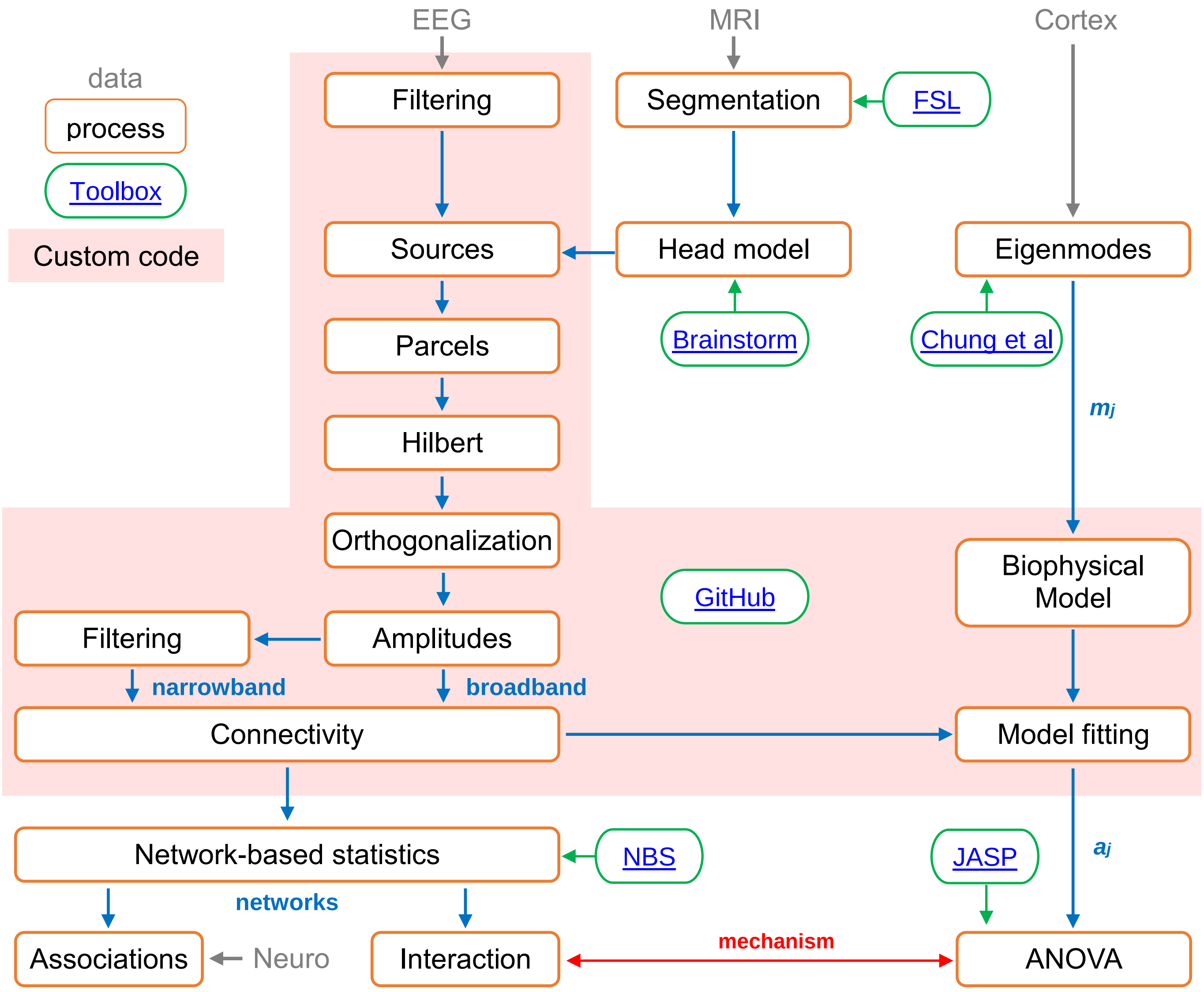

Supplementary Fig. 9. *Overview of the analytical pipeline.* This schematic shows the major processing stages (orange boxes) of the data processing. Software packages that were used at each stage are indicated in the green boxes (with hyperlinks to the corresponding download sites).
