# Supplementary material for: Large-scale brain modes reorganize between infant sleep states and carry prognostic information for preterms
Source: Nat Commun. 2019 Jun 13;10:2619. doi: 10.1038/s41467-019-10467-8 (PMC6565810; doi:10.1038/s41467-019-10467-8)
Supplement: Supplementary file 3 — Reporting Summary [file 41467_2019_10467_MOESM3_ESM.pdf]

nature research

Corresponding author(s): NCOMMS-18-38206B
Anton Tokariev

Last updated by author(s): Apr 21, 2019

Reporting Summary

Nature Research wishes to improve the reproducibility of the work that we publish. This form provides structure for consistency and transparency in reporting. For further information on Nature Research policies, see Authors & Referees and the Editorial Policy Checklist.

Statistics

For all statistical analyses, confirm that the following items are present in the figure legend, table legend, main text, or Methods section.

n/a	Confirmed	
☐	☒	The exact sample size (*n*) for each experimental group/condition, given as a discrete number and unit of measurement
☐	☒	A statement on whether measurements were taken from distinct samples or whether the same sample was measured repeatedly
☐	☒	The statistical test(s) used AND whether they are one- or two-sided<br>*Only common tests should be described solely by name; describe more complex techniques in the Methods section.*
☐	☒	A description of all covariates tested
☐	☒	A description of any assumptions or corrections, such as tests of normality and adjustment for multiple comparisons
☐	☒	A full description of the statistical parameters including central tendency (e.g. means) or other basic estimates (e.g. regression coefficient) AND variation (e.g. standard deviation) or associated estimates of uncertainty (e.g. confidence intervals)
☐	☒	For null hypothesis testing, the test statistic (e.g. *F*, *t*, *r*) with confidence intervals, effect sizes, degrees of freedom and *P* value noted<br>*Give P values as exact values whenever suitable.*
☒	☐	For Bayesian analysis, information on the choice of priors and Markov chain Monte Carlo settings
☒	☐	For hierarchical and complex designs, identification of the appropriate level for tests and full reporting of outcomes
☐	☒	Estimates of effect sizes (e.g. Cohen's *d*, Pearson's *r*), indicating how they were calculated

Our web collection on statistics for biologists contains articles on many of the points above.

Software and code

Policy information about availability of computer code

Data collection	EEG data were collected with the commercial NicOne (Cardinal Healthcare/Natus, USA) and Cognitrace (EANT B.V., Enschede, The Netherlands) recorders, using their in-built, commercially available recording software. EEG caps (sintered Ag/AgCl electrodes; Waveguard, ANT-Neuro, Germany) had 19 or 28 scalp electrodes positioned according to the International 10–20 standard.<br><br>For extremely preterm infants, neurodevelopment was assessed at two years of corrected age using the structured Griffiths Mental Developmental Scales.
Data analysis	Initial review of the EEG data was performed using commercial review software of the EEG manufacturer. The actual data analysis was performed using custom Matlab codes (version R2016b) and open analytical toolboxes:<br><br>- Segmentation of infant structural scans (Magnetic Resonance Imaging, MRI) was performed using FSL: http://fsl.fmrib.ox.ac.uk/fsl/fslwiki (Smith et al. 2004)<br>- The head model was computed using the openMEEG package: http://openmeeg.github.io (Gramfort et al. 2010)<br>- Sources were computed using dSPM algorithm in Brainstorm: http://neuroimage.usc.edu/brainstorm (Tadel et al. 2011)<br>- Statistical comparison of whole-brain functional connectivity was performed with the Network-Based Statistic: https://www.nitrc.org/projects/nbs (Zalesky et al., 2010)<br>- Cortical connectivity patterns were visualized using BrainNet Viewer: http://www.nitrc.org/projects/bnv (Xia et al. 2013)<br>- Statistical analyses were performed using standard and custom Matlab functions and the JASP software: https://jasp-stats.org/download<br><br>We provide all custom Matlab codes that were used in this work at: https://github.com/babyEEG/Infant-Sleep<br>We also show the entire analytic pipeline, together with download links (to the original and custom toolkits) at the Supplementary Fig.S9.

For manuscripts utilizing custom algorithms or software that are central to the research but not yet described in published literature, software must be made available to editors/reviewers. We strongly encourage code deposition in a community repository (e.g. GitHub). See the Nature Research guidelines for submitting code & software for further information.

Data

Policy information about availability of data

All manuscripts must include a data availability statement. This statement should provide the following information, where applicable:

- Accession codes, unique identifiers, or web links for publicly available datasets
- A list of figures that have associated raw data
- A description of any restrictions on data availability

The original (raw) EEG data will be made available upon request to the authors (S.V.). The use of this dataset in further scientific work will require a data sharing agreement with Helsinki University Hospital. Processed data, such as connectivity matrices, can be made available upon request.

Field-specific reporting

Please select the one below that is the best fit for your research. If you are not sure, read the appropriate sections before making your selection.

☒ Life sciences ☐ Behavioural & social sciences ☐ Ecological, evolutionary & environmental sciences

For a reference copy of the document with all sections, see nature.com/documents/nr-reporting-summary-flat.pdf

Life sciences study design

All studies must disclose on these points even when the disclosure is negative.

Sample size	This was an exploratory study that used retrospective datasets with no prior determination of sample size. EEG data were available for two groups of infants: extremely preterm (N = 46) and full-term healthy controls (N = 67). EEG data in both groups were acquired at term-equivalent age of 41.1 ± 2 (median ± interquartile range, IQR) weeks. Some data within these cohorts have been previously published (Omidvarnia et al. 2014; Tokariev et al. 2018). The scope of this previous work was independent to the current study.
Data exclusions	For the analysis we selected infants that had 5-min-long artifact-free EEG in each of two distinct vigilance states: active sleep and quiet sleep (see Tokariev et al., 2016; Tokariev et al., 2018). This led to the following group sizes: extremely preterm (N = 42) and full-term healthy controls (N = 52).
Replication	This is a unique clinical data set of high density EEG from preterm and matched full term infants. To our knowledge, there are no available replication datasets. To ensure that our findings can be reliably reproduced, functional connectivity matrices and code will be available by request.
Randomization	This is not a randomized treatment study.
Blinding	Blinding is not relevant to this study because it is not a randomized treatment study.

Reporting for specific materials, systems and methods

We require information from authors about some types of materials, experimental systems and methods used in many studies. Here, indicate whether each material, system or method listed is relevant to your study. If you are not sure if a list item applies to your research, read the appropriate section before selecting a response.

Materials & experimental systems

n/a	Involved in the study	
☒	☐	Antibodies
☒	☐	Eukaryotic cell lines
☒	☐	Palaeontology
☒	☐	Animals and other organisms
☐	☒	Human research participants
☒	☐	Clinical data

Methods

n/a	Involved in the study	
☒	☐	ChIP-seq
☒	☐	Flow cytometry
☒	☐	MRI-based neuroimaging

Human research participants

Policy information about studies involving human research participants

Population characteristics	We analyzed two groups of subjects: extremely preterm (N = 42), born at gestational age of 26.6 ± 1.6 [median ± interquartile range, IQR] weeks; 12 girls) and full-term healthy controls (N = 52, born at gestational age of 40.4 ± 1.8 [median ± IQR] weeks; 19 girls). EEG data in both groups were acquired at term-equivalent age of 41.1 ± 2 (median ± IQR) weeks.
Recruitment	Participation in the study was voluntary. The preterm cohort was recruited over a period of approximately two years. After

Recruitment	parental consent, preterm infants were excluded if their clinical condition was not stable. The healthy control cohort was recruited via media advertising.
Ethics oversight	The study design and procedures have been approved by the Ethics Committee of the Helsinki University Central Hospital (Finland). Informed written consent was received from a guardian before inclusion of an infant into the study.

Note that full information on the approval of the study protocol must also be provided in the manuscript.
